# Study of Ocular Fundus Changes in Pregnancy-Induced Hypertension and Visual Outcomes After Delivery

**DOI:** 10.7759/cureus.117038

**Published:** 2026-09-27

**Authors:** Kuldeep Chaudhary, Marianus Deepak Lakra, Sindhu Kumari, Pinki Patel, Rajkumari Meena, Archana Kumari

**Affiliations:** 1 Department of Ophthalmology, Rajendra Institute of Medical Sciences, Ranchi, IND; 2 Department of Obstetrics and Gynecology, Rajendra Institute of Medical Sciences, Ranchi, IND

**Keywords:** hypertensive retinopathy, ocular fundus changes, preeclampsia, pregnancy-induced hypertension, visual outcomes

## Abstract

Background: Pregnancy-induced hypertension (PIH), a leading cause of maternal and fetal morbidity and mortality, often results in ocular involvement, most notably hypertensive retinopathy. The aim of this prospective research was to investigate the types and prevalence of ocular fundus changes in PIH patients and to assess their visual outcomes following delivery.

Methods: In a prospective observational study, 400 pregnant women with a clinical diagnosis of PIH were included. Everyone who participated received a thorough ocular checkup that included fundoscopy and an evaluation of their visual acuity. The Keith-Wagener-Barker classification was used to grade the retinal alterations. To record the resolution of retinal abnormalities and visual recovery, patients were monitored at one, four, and 12 weeks after giving birth.

Results: Ocular fundus changes were observed in 179 women (44.8%). The most prevalent finding was retinal hemorrhages, noted in 288 patients (72%), followed by retinal exudates in 195 patients (48.8%). The presence and severity of hypertensive retinopathy correlated significantly with the grade of PIH. Following delivery, visual acuity showed progressive improvement. At one week, 20% of patients had severe visual impairment, which decreased to 1.3% by 12 weeks. Notably, 80% of patients regained normal vision (6/6) at the 12-week follow-up.

Conclusion: Ocular fundus changes are a frequent complication of PIH, and their presence is a reliable indicator of systemic disease severity. The study demonstrates that visual impairment associated with PIH is largely transient, with the vast majority of patients experiencing significant visual recovery within three months of delivery. These findings provide crucial evidence for patient counseling and underscore the value of routine ocular assessment in the management of PIH.

## Introduction

The condition known as pregnancy-induced hypertension (PIH) occurs when a woman's blood pressure, which was previously normal, increases to ≥140/90 mmHg after 20 weeks of pregnancy. Preeclampsia, eclampsia, and gestational hypertension are among them. These conditions continue to be a leading source of disease and mortality for both women and newborns, affecting between 7% and 10% of pregnancies globally [1]. A more severe form of PIH known as preeclampsia is characterized by elevated blood pressure and urine protein. It may occasionally develop into eclampsia, which is typified by unconsciousness or convulsions. Damage to the inner lining of blood vessels, elevated vascular resistance, and decreased blood flow to vital organs, such as the eyes, are the primary alterations causing PIH [2,3].

Eye involvement is common in PIH and occurs in about one-third to more than half of affected women. The most frequent changes are seen in the retina and are called hypertensive retinopathy. These include narrowing of retinal arteries, cotton wool spots, small hemorrhages, and exudates. The severity of these changes often reflects the severity of hypertension. A simple eye examination using fundoscopy is a quick, non-invasive way to detect these abnormalities [3,4]. Many of the vascular changes improve after delivery, but in severe cases, complications like retinal detachment, central retinal artery occlusion, or ischemia of the choroid and optic nerve may cause permanent vision loss [5].

Patients may complain of blurred vision, flashing lights, dark spots, or temporary loss of vision. In rare cases (1-3%), permanent blindness can occur [5]. These findings show the importance of routine eye examination in women with PIH, not only to assess their eye health but also to get an idea of how severe their systemic disease might be [6]. Retinal changes may also reflect poor placental blood supply, which directly affects the baby. The problem of PIH is especially severe in developing countries like India, where it complicates around 15% of pregnancies, particularly in first-time mothers [6,7]. Limited access to antenatal care and poor health facilities make the outcomes worse, and eclampsia remains an important cause of maternal death, with mortality rates ranging from 2% to as high as 23% in some studies [8].

Although several studies have described ocular changes in PIH, there is still limited data from prospective studies that follow patients after delivery to see whether their visual problems recover or persist. Most available studies are retrospective and report variable rates of retinopathy, but they often do not assess postpartum outcomes [9-11]. Since delivery is the main treatment for PIH and many of the eye changes are reversible, it is important to know which patients are likely to recover vision and which ones are at risk of permanent loss [12]. The current study was planned to examine the types of ocular fundus changes seen in patients with PIH and to follow them after delivery to study their visual outcomes. The findings are expected to add to existing knowledge and help improve clinical care for both mothers and newborns.

## Materials and methods

Research design and site

The present research was conducted at the Regional Institute of Medical Sciences (RIMS), Ranchi, Jharkhand, India, in the Department of Ophthalmology in cooperation with the Department of Obstetrics and Gynecology after obtaining approval from the institute's Institutional Ethics Committee (approval number: 157/IEC, RIMS; date: May 10, 2024). The time duration of this work was August 2023 to February 2025.

Study cohort

The required sample size was calculated as 389, based on a reported prevalence of PIH of 6.5% among the Indian population. To enhance study power, a total of 400 patients were included [8].

Participant selection

Eligible participants included pregnant women with a clinical diagnosis of PIH who were admitted to the Department of Obstetrics and Gynecology, attended the antenatal clinic, or were in the labor room. Women who were willing to give written informed consent, were between the ages of 18 and 35, and had gestational ages longer than 20 weeks were sought. Patients who had a history of trauma, surgery, or laser treatment or who had ocular diseases, such as glaucoma, cataracts, corneal opacity, diabetes mellitus, hypertension, renal disease, or cardiovascular disease, were not included. Systemic diseases such as anemia, human immunodeficiency virus (HIV), connective tissue disorders, vasculitis, leukemia, and cancer were also excluded.

Maternal assessment

All enrolled women underwent a detailed obstetric and medical history, along with general physical and systemic examination. Blood pressure was recorded and classified according to the severity of PIH. Laboratory investigations included complete blood count, liver and renal function tests, and urine protein estimation.

Ocular assessment

Visual acuity was assessed using Snellen's chart. For patients who were unable to undergo standard Snellen visual acuity assessment because they were confined to bed, visual acuity was assessed using the counting-fingers method at an appropriate distance and recorded accordingly. The anterior segment was examined with torchlight and slit-lamp biomicroscopy. Pupillary dilation was achieved using tropicamide 0.8% with phenylephrine 5%. Fundus examination was performed using both direct and indirect ophthalmoscopy, and fundus photographs were obtained with a Volk InView fundus camera (Volk Optical, Inc., Mentor, Ohio, United States). Optical coherence tomography (OCT) was carried out selectively to evaluate macular changes. Retinal abnormalities were graded according to the Keith-Wagener-Barker (KWB) classification [4,11]. Fundus photography and OCT were performed selectively when clinically indicated to document or further evaluate ocular findings. These investigations were not performed uniformly in all participants and were therefore not included as separate outcome measures in the present analysis.

Postpartum assessment

All patients were followed up after delivery at one, four, and 12 weeks in the Ophthalmology outpatient department. Resolution or persistence of retinal changes, along with visual outcomes, were recorded.

Data analysis

Microsoft Excel (Microsoft Corporation, Redmond, Washington, United States) was used to enter the data, and IBM SPSS Statistics for Windows, Version 21.0 (IBM Corp., Armonk, New York, United States) was used for analysis. Frequencies and percentages were used to represent categorical data, whereas mean±standard deviation was used to represent continuous variables. For variables that were continuous, Student's t-test was employed, whereas the chi-squared test was utilized for categorical variables. P-values less than 0.05 were regarded as statistically significant.

## Results

Demographic and obstetric profile

This prospective study included 400 women diagnosed with PIH, all of whom were between the ages of 18 and 40. Around 41.5% of the population was between the ages of 26 and 30, and 38.8% were between the ages of 21 and 25. Just 2.5% of the patients were older than 35. Fifty-four percent of the women were primigravida, and 46% were multigravida. Thirty-three percent of the women gave birth before 37 weeks, while 34% of the women gave birth between 37 and 38 weeks. Just 8% of the women carried their pregnancies over 40 weeks. The demographic and clinical details of the 400 pregnant women with PIH who were part of the trial are compiled in Table 1.

**Table 1:** Demographic and clinical characteristics of pregnancy-induced hypertension patients (n=400)

Parameter	Category	n (%)
Age (years)	≤20	16 (4)
	21-25	155 (38.8)
	26-30	166 (41.5)
	31-35	53 (13.3)
	36-40	10 (2.5)
Gravida status	Primigravida	216 (54)
	Multigravida	184 (46)
Gestational age (weeks)	<37	132 (33)
	37-38	136 (34)
	39-40	100 (25)
	>40	32 (8)

Obstetric management

The decision regarding the continuation or termination of pregnancy was made by the treating obstetric team based on the severity of PIH, gestational age, maternal clinical condition, and fetal status. In the three cases requiring a specific decision regarding pregnancy termination or continuation, the management plan was individualized according to the maternal and fetal indications documented at the time of admission. The indication for termination, where performed, and the clinical rationale for continued pregnancy, where applicable, were recorded.

Severity of PIH and fundus changes

A total of 400 women with PIH were enrolled. The mean age of the study population was 24.3±2.8 years, and just over half (54%) were primigravidae. Most deliveries occurred between 37 and 38 weeks of gestation. Blurring of vision was the most common ocular complaint, reported by 45% of patients, while 13.3% presented with convulsions.

The distribution of disease severity showed that 190 patients (47.5%) had non-severe PIH, 193 (48.3%) had severe PIH, and 17 (4.3%) were classified as very severe PIH. Ocular fundus changes were documented in 179 women (44.8%), while 221 (55.3%) showed no abnormalities.

Among the 179 women with fundus changes, hypertensive retinopathy was graded according to the KWB classification. Mild hypertensive retinopathy (KWB grade I) was observed in 144 patients (80.4%), moderate hypertensive retinopathy (KWB grade II) in 25 (14%), and severe hypertensive retinopathy (KWB grade III) in 10 (5.6%). Fundus involvement increased with increasing severity of PIH, being observed in 60 of 190 women (31.6%) with non-severe PIH, 102 of 193 women (52.8%) with severe PIH, and all 17 women (100%) with very severe PIH. Higher-grade hypertensive retinopathy was more frequently observed among women with severe and very severe PIH (χ² test; p<0.001). The distribution of patients according to PIH severity, fundus changes, and KWB grade is summarized in Table 2.

**Table 2:** Distribution of patients by PIH grade, grade, and fundus changes (n=400) PIH: pregnancy-induced hypertension; HR: hypertensive retinopathy; KWB: Keith-Wagener-Barker

PIH severity	Number of patients	Percentage (%)	Fundus changes present, n (%)	HR grade distribution
Non-severe PIH	190	47.5	60 (31.6)	Mild (KWB grade I): 55 (91.7%); moderate (KWB grade II): 4 (6.7%); severe (KWB grade III): 1 (1.7%)
Severe PIH	193	48.3	102 (52.8)	Mild (KWB grade I): 82 (80.4%); moderate (KWB grade II): 16 (15.7%); severe (KWB grade III): 4 (3.9%)
Very severe PIH	17	4.3	17 (100)	Mild (KWB grade I): 7 (41.2%); moderate (KWB grade II): 5 (29.4%); severe (KWB grade III): 5 (29.4%)
Total	400	100	179 (44.8)	Mild (KWB grade I): 144 (80.4%); moderate (KWB grade II): 25 (14%); severe (KWB grade III): 10 (5.6%)

Correlation of systemic severity with ocular changes

Among the 400 women with PIH, ocular fundus abnormalities were observed in 179 patients (44.8%). The most common retinal lesions were hemorrhages, which were observed in 141 left eyes (35.3%) and 147 right eyes (36.8%). Additional observations included vascular attenuation in 23 left eyes (5.8%) and 25 right eyes (6.3%) and retinal exudates in 92 left eyes (23%) and 103 right eyes (25.8%). Papilledema, reported as total cases, was present in 56 patients (14%), while serous retinal detachment was identified in three patients (0.8%). These findings reflect the classical spectrum of hypertensive retinopathy changes associated with PIH. Table 3 details the spectrum of ocular fundus changes observed in the 400 patients with PIH.

**Table 3:** Spectrum of ocular fundus changes in pregnancy-induced hypertension patients (n=400) Ocular findings are reported separately for each eye. The asterisk (*) indicates that papilledema (grades 1-2) was recorded as a patient-level finding and was not analyzed separately by right eye and left eye; therefore, statistical comparison between the right and left eyes was not applicable.

Finding	Right eye n (%)	Left eye n (%)	Total n (%)	χ² (p-value)
Vessel attenuation	25 (6.3)	23 (5.8)	48 (12)	χ²=0.09 (p=0.766)
Retinal hemorrhages	147 (36.8)	141 (35.3)	288 (72)	χ²=0.20 (p=0.659)
Retinal exudates	103 (25.8)	92 (23)	195 (48.8)	χ²=0.82 (p=0.365)
Papilledema (grades 1-2)*	-	-	56 (14)	N/A
Serous retinal detachment	-	-	3 (0.8)	N/A

Visual outcomes after delivery

Visual acuity improved progressively following delivery. At one week, only 50 (12.5%) of patients achieved normal vision, while 80 (20%) continued to have severe impairment (≤6/36). By four weeks, 200 (50%) of patients had regained normal vision, with severe impairment falling to 10 (2.5%). At 12 weeks, 320 (80%) of patients demonstrated complete recovery, 60 (15%) had mild residual impairment, 15 (3.8%) had moderate impairment, and five (1.3%) continued to have severe visual loss (Table 4).

**Table 4:** Visual outcomes during postpartum follow-up (n=400) The dagger (‡) indicates that the reported chi-squared test represents the overall comparison of visual acuity distributions across the three postpartum follow-up time points (one, four, and 12 weeks), with p<0.001 indicating a statistically significant difference.

Visual acuity	1 week n (%)	4 weeks n (%)	12 weeks n (%)	χ² (p-value)
Normal (6/6)	50 (12.5)	200 (50)	320 (80)	χ²=451.83; df=6; p<0.001‡
Mild (6/9-6/12)	150 (37.5)	150 (37.5)	60 (15)	χ²=451.83; df=6; p<0.001‡
Moderate (6/18-6/24)	120 (30)	40 (10)	15 (3.8)	χ²=451.83; df=6; p<0.001‡
Severe (≤6/36)	80 (20)	10 (2.5)	5 (1.3)	χ²=451.83; df=6; p<0.001‡

Visual recovery was significantly faster in preeclampsia compared to eclampsia. Among preeclampsia patients, nearly all had regained normal acuity by 12 weeks, whereas in eclampsia, a proportion continued to experience mild-to-moderate impairment.

## Discussion

The current research tracked the visual outcomes of women with PIH after delivery. A range of ocular fundus abnormalities were also studied in these individuals. Our results offer useful longitudinal information on visual recovery and support the documented association between the degree of PIH and the existence of retinal impairments.

Our study found that ocular fundus changes were present in 44.8% of the PIH patients, a finding consistent with previous literature, which reports a wide range of prevalence from one-third to over half of affected women. The most common ocular finding was retinal hemorrhages (72%), followed by retinal exudates (48.8%). This pattern of findings, along with vessel attenuation, reflects the classic manifestations of hypertensive retinopathy. A significant association was observed between the clinical severity of PIH and the severity of hypertensive retinopathy. The retinal findings were graded according to the KWB classification, supporting the use of fundus examination as a non-invasive assessment of hypertensive retinal involvement [3,4]. The presence of these findings underscores the systemic vascular damage caused by PIH, which is mirrored in the delicate retinal circulation.

A key strength of this study is its prospective design and the extended follow-up period, which allowed us to track visual outcomes after delivery. We observed a remarkable and progressive improvement in visual acuity. While a significant portion of patients had severe visual impairment at one week postpartum (20%), this number decreased dramatically to just 1.3% by 12 weeks. Most notably, 80% of patients had regained normal vision by the 12-week mark. This is a crucial finding as it provides reassurance that the majority of vision loss associated with PIH is temporary and reversible. The swift recovery highlights that delivery, which resolves the underlying cause of the hypertension, is the most effective treatment for both the systemic and ocular manifestations of the disease.

Our findings on visual recovery are particularly relevant for patient counseling. While the initial ocular symptoms and fundus changes can be alarming, our data suggest that for most patients, these are transient. This aligns with a previous study that also noted the reversibility of PIH-related eye changes [12]. The persistent visual impairment in a small minority of patients, particularly those with eclampsia, underscores the importance of close monitoring in the most severe cases. This remaining visual loss is likely related to more permanent damage, such as from choroidal or optic nerve ischemia, which can occur in the most severe forms of the disease [5].

Several previous studies have similarly demonstrated a strong association between PIH and retinal vascular changes, reinforcing the clinical value of fundus examination in these patients. Tadin et al. reported that hypertensive retinopathy was present in approximately 45% of women with preeclampsia and that the severity of retinal changes was directly correlated with the severity of systemic hypertension, highlighting the usefulness of retinal findings as an indicator of systemic vascular involvement [13]. Likewise, Karki et al. observed retinal abnormalities in nearly half of preeclamptic patients and emphasized that retinal examination serves as a reliable, non-invasive method to assess disease severity and monitor progression [14]. These findings support our results and further confirm that retinal evaluation can provide important prognostic information, helping clinicians identify high-risk patients and guide appropriate monitoring and management strategies.

In conclusion, this study confirms that hypertensive retinopathy is a common finding in PIH and that its severity is directly linked to the severity of the systemic disease. More importantly, it demonstrates that visual recovery is a rapid and highly favorable process for the vast majority of patients after delivery. These results advocate for routine eye examinations as a valuable part of the assessment for PIH and provide a strong evidence base for reassuring patients about their visual prognosis. The findings also highlight the need for continued vigilance and care for the small group of patients who do not experience full visual recovery.

Limitations

Despite its prospective design and adequate sample size, the present study has certain limitations. First, the study was conducted at a single tertiary care center, which may limit the generalizability of the findings to other populations, especially those in primary or rural healthcare settings. Second, although fundus examination was performed using standard clinical methods, advanced imaging modalities such as OCT and fundus photography were used selectively rather than uniformly in all patients, which may have led to the under-detection of subtle retinal changes. Third, the follow-up period was limited to 12 weeks postpartum; therefore, long-term visual outcomes and the possibility of permanent retinal damage beyond this period could not be assessed. Fourth, the study did not include a control group of normotensive pregnant women, which limits direct comparison and assessment of relative risk. Finally, systemic factors such as duration of hypertension, biochemical markers, and detailed fetal outcomes were not analyzed in correlation with retinal findings, which could have provided additional insights into disease severity and prognosis.

## Conclusions

This study confirms that ocular fundus changes are a common manifestation of PIH and that their severity is directly correlated with the grade of the systemic disease. More importantly, our prospective findings demonstrate that visual impairment associated with PIH is a largely reversible condition. The rapid and significant visual recovery observed in the majority of patients after delivery provides a strong evidence base for reassuring mothers about their visual prognosis.

These results highlight two key clinical takeaways. First, fundoscopy serves as a valuable, non-invasive tool for assessing the severity of PIH. Second, a proactive approach to patient counseling, emphasizing the high probability of visual recovery, is essential for alleviating patient anxiety. Our findings add to the limited prospective data available on this topic and advocate for continued vigilance and follow-up, particularly for the small subset of patients who may not experience full visual recovery.
